# Effectiveness and Safety of Belimumab in Chinese Lupus Patients: A Multicenter, Real-World Observational Study

**DOI:** 10.3390/biomedicines11030962

**Published:** 2023-03-21

**Authors:** Fangfang Sun, Huaxiang Wu, Zitao Wang, Tong Wu, Xue Wu, Jie Chen, Danting Zhang, Chunde Bao, Nan Shen, Lijun Wu, Jing Zhu, Shuang Ye

**Affiliations:** 1 Department of Rheumatology, Renji Hospital, School of Medicine, Shanghai Jiaotong University, Shanghai 201112, China; 2 Department of Rheumatology, The Second Affiliated Hospital Zhejiang University School of Medicine, Hangzhou 310009, China; 3 Department of Rheumatology, Sichuan Provincial People’s Hospital, University of Electronic Science and Technology of China, Chengdu 610072, China; 4 Department of Rheumatology, People’s Hospital of Xinjiang Uygur Autonomous Region, Urumqi 830001, China; 5 Xinjiang Clinical Research Center for Rheumatoid Arthritis, Urumqi 830001, China

**Keywords:** systemic lupus erythematosus (SLE), SLE Responder Index 4 (SRI-4), Lupus Low Disease Activity State (LLDAS), remission, disease flare

## Abstract

**Objective**: The effectiveness and safety of belimumab in Chinese lupus patients with different disease activities were investigated in a real-world setting. **Method**: Patients who received 10 mg/kg belimumab intravenously on weeks 0, 2, and 4, and then every 4 weeks on a background of standard-of-care (SoC) therapy and had a follow-up of more than 6 months were enrolled from four centers in China. They were stratified according to the Safety of Estrogens in Lupus Erythematosus National Assessment-SLE Disease Activity Index (SELENA-SLEDAI) score at baseline as the moderate/severe (SELENA-SLEDAI > 6) or mild subgroups (SELENA-SLEDAI ≤ 6). Attainment of the Lupus Low Disease Activity State (LLDAS) or remission on treatment was analyzed in all patients. The SLE Responder Index 4 (SRI-4) and SELENA-SLEDAI Flare Index (SFI) were evaluated for patients with moderate/severe disease and mild disease, respectively. Patients in the control arm with SoC alone from previous metformin lupus trials were selected by propensity score matching (PSM) as the reference group. **Results**: 224 SLE patients with a mean follow-up of 11.7 months receiving belimumab were enrolled in this observational study, of which 126 and 98 were in the moderate/severe and mild subgroup, respectively. At 12 months, 54.76% of the patients attained LLDAS and 28.57% attained remission. Lower daily prednisone at baseline were independently associated with 12-month LLDAS. Further, 87% of the subgroup with moderate/severe disease achieved SRI-4 at 12 months and a high SLEDAI at baseline was its predictive factor. For the mild subgroup, a reduced flare rate was observed compared with PSM reference (17.5%, vs. 38.6%, *p* = 0.021). Infection events, particularly viral infections and pneumonia were recorded in 7 and 6 patients, respectively. **Conclusion**: Our real-world data supported the effectiveness and safety of belimumab in Chinese lupus patients.

## 1. Introduction

Systemic lupus erythematosus (SLE) is a heterogeneous systemic autoimmune disease with different disease activity statuses during its time course [1]. The first FDA-approved biological agent for active SLE, belimumab, is a human monoclonal antibody targeting B-lymphocyte stimulator (BLyS), which is a key molecule involved in SLE [2]. It has been reported in three cardinal phase III trials including BLISS-52, BLISS-76, and BLISS-North East Asia (NEA) that 10 mg/kg belimumab was effective in reducing disease activity in terms of SLE Responder Index-4 (SRI-4) in different populations [3,4,5]. Furthermore, the NEA trial identified that patients receiving belimumab had a significantly lower risk of major disease flare than those in the placebo arm [3]. Regarding the treat-to-target aim in lupus patients, post-hoc analysis of two BLISS trials demonstrated that belimumab was helpful in attaining the Lupus Low Disease Activity State (LLDAS) [6]. Additionally, the effectiveness of belimumab has also been validated in many Western countries in the real-world settings, such as in OBSErve (Evaluation Of use of Belimumab in clinical practice SEttings) and BeRLiSS (BElimumab in Real LIfe Setting Study) cohorts [7,8,9,10,11]. The evidence is solid that belimumab helps control disease activity, sparing glucocorticoid exposure and reducing damage accrual.

The rationale to initiate the current study is that real-world data among Chinese SLE patients to address the effectiveness and safety of belimumab are still lacking. Data are insufficient for specific patient groups, especially those with lower disease activities (e.g., SLEDAI < 6). Frequent disease flares followed by organ damage can still be observed in this population [12], which is an important clinical problem left unsolved. Whether belimumab could be a maintenance therapy in lupus patients with low-grade disease activity is another important issue we want to address in this real-world study. Moreover, to better implement treat-to-target strategy in practice by using belimumab, for those with low grade disease in particular, is yet to be exploited. Thus, we conducted this multicenter, real-world study that enrolled SLE patients from the east to west of China mainland, with a “silk road” pattern, which included both Han and non-Han ethnicities.

## 2. Patients and Methods

This multicenter, real-world study was conducted in four referral centers (Renji Hospital, Shanghai Jiaotong University School of Medicine, Center 1; The Second Affiliated Hospital, Zhejiang University School of Medicine, Center 2; Sichuan Provincial People’s Hospital, University of Electronic Science and Technology of China, Center 3; People’s Hospital of Xinjiang Uygur Autonomous Region, Center 4). This investigator-initiated, non-industry sponsored observational study was approved by the central ethics committee of Renji Hospital, Shanghai Jiaotong University School of Medicine (KY2021-050) with informed consent obtained from all participants. All methods were performed in accordance with the relevant guidelines and regulations.

## 3. Inclusions and Exclusions

Patients from our centers who met the 1997 American College of Rheumatology (ACR) criteria for SLE [13] and received belimumab on the background of standard-of-care (SoC) were included from October 2019 (the sino-FDA approval date for belimumab) to July 2021. Belimumab was given intravenously at the dose of 10 mg/kg on weeks 0, 2, and 4, and then every 4 weeks. Patients with exposure to other B cell-targeted therapies within 6 months before initiation of belimumab or with a follow-up of less than 6 months since the first dose were not included in the analysis. Patients with active infections, current pregnancy or breast feeding, or with a history of malignancy within the last 5 years were excluded from the study.

## 4. Evaluations

Demographic characteristics including ethnicity, clinical manifestations, laboratory tests, serological results (complements and anti-ds-DNA positivity), disease activity, and treatments (daily dosage of prednisone, hydroxychloroquine (HCQ) and immunosuppressive agents (IS)) were collected at baseline, and every three months thereafter. Adverse events and reasons of drug discontinuation were also documented. Disease activity was evaluated by the Safety of Estrogens in Lupus Erythematosus National Assessment-SLE Disease Activity Index (SELENA-SLEDAI) and Physician’s Global Assessment of disease activity (PGA).

LLDAS/remission attainment was assessed in the intention-to-treat population. LLDAS was defined as SLEDAI ≤ 4, daily prednisone ≤ 7.5 mg, no activity in any major organ or no new disease activity features, and PGA ≤ 1. Remission on treatment was defined as clinical SLEDAI score = 0 and PGA < 0.5 with prednisone ≤ 5 mg/day, HCQ and IS as maintenance [14,15].

Additional endpoints included changes in complements, anti-ds-DNA positivity, SLEDAI score, PGA, and daily prednisone. Due to the diversity of the anti-ds-DNA autoantibody assays in different centers, including multiplex-based immunoassay, Farr radioimmunoassay, ELISA and *Crithidia Luciliae* indirect immunofluorescence test [16], only its positivity was evaluated.

## 5. Subgroups

Patients were divided by baseline SLEDAI score into moderate/severe (SLEDAI > 6) and mild disease (SLEDAI ≤ 6) subgroups. SRI-4 was applied as the effectiveness endpoint for the moderate/severe subgroup, while disease flares defined by the modified SELENA-SLEDAI Flare Index (SFI) [17,18] were evaluated in the mild subgroup. For comparison of disease flares, we introduced subjects in the control arm treated with SoC alone as reference group from our previous metformin randomized trials [12,19], which were the only two existing and similarly designed trials to address the lupus flare prevention outcome according to SFI in lupus patients with low grade disease activity (SLEDAI ≤ 6). The possible confounders were further evened out by propensity score matching between patients in mild group treated with belimumab in this real-world study and reference group treated with SoC alone from trials.

## 6. Statistical Methods

Continuous variables are presented as the mean ± SD and categorical data are listed as numbers (percentages). Missing data at baseline were addressed by multiple imputation, and the following missing data were imputed using the last observation carried forward method. Baseline parameters were compared using the Mann–Whitney U test for continuous variables or chi-square’s test with Yates’ correction for categorical data. Endpoints during follow-up were compared with baseline using the Wilcoxon signed rank test or McNemar’s test. Binary logistic regression analysis was applied to find independent predictors of 12-month SRI-4 response for the moderate/severe disease subgroup and 12-month LLDAS attainment for all the patients adjusting possible confounders. Relative subgroup analyses were performed according to study center, and ethnicity and baseline characteristics selected by clinical significance. Propensity score matching was used to identify matched populations adjusting for age, disease duration, SLEDAI, and prednisone dose. Flare-free survivals were analyzed by Kaplan–Meier curves. *p*-values < 0.05 were considered significant. Statistical analysis was conducted with SPSS software (version 26.0), GraphPad software (version 8.0), and R software (version 3.6.3).

## 7. Results

**Figure 1:**
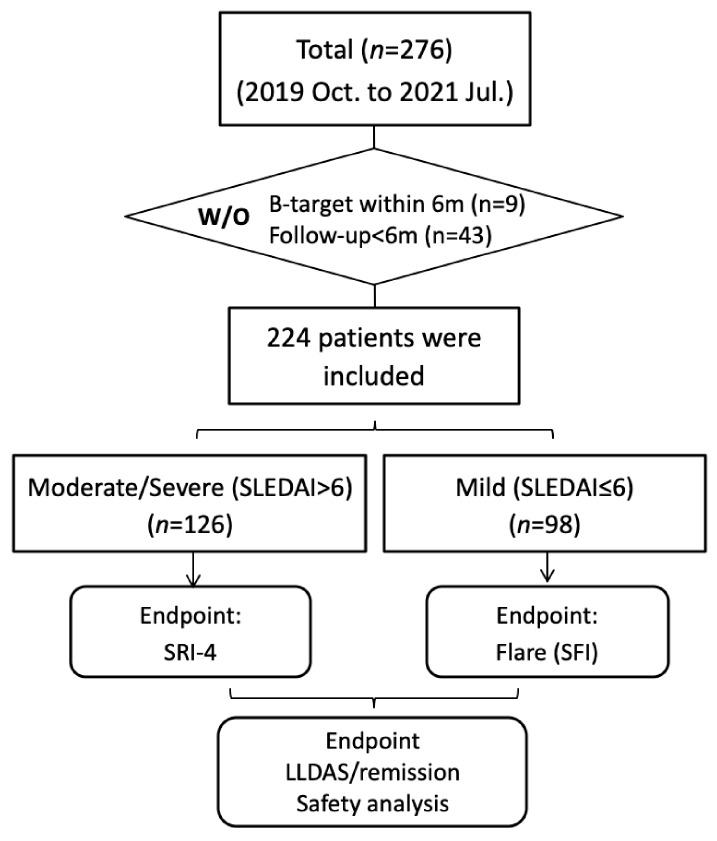
Flow chart of the study.

A total of 224 patients who received belimumab treatment were eligible for the analysis in this real-world study with a mean follow-up of 11.7 months (6 months to 27 months). The detailed flow chart is presented in Figure 1 and the baseline characteristics of these patients are listed in Table 1 (detailed data by individual centers are presented in Table S1). They had a mean age of 34.09 ± 10.91 years, of which 95.1% were female. A total of 94.2% were of Han ethnicity, and 5.8% were Uygur ethnicity. 33.5% patients had manifestations of lupus nephritis. Their baseline prednisone was 23.97 ± 19.68 mg/day, and their baseline SLEDAI score was 7.72 ± 4.46. The positive rate of anti-ds-DNA was 75.4% at baseline and low complement 3 and 4 were observed in 66.1% and 45.1% of the patients, respectively. All the patients were taking SoC, of which 88.4% (*n* = 198) and 59.8% (*n* = 134) of these patients were taking HCQ and IS, respectively. Mycophenolate, calcineurin inhibitors (CNIs) and cyclophosphamide were the most commonly used ISs.

**Figure 2:**
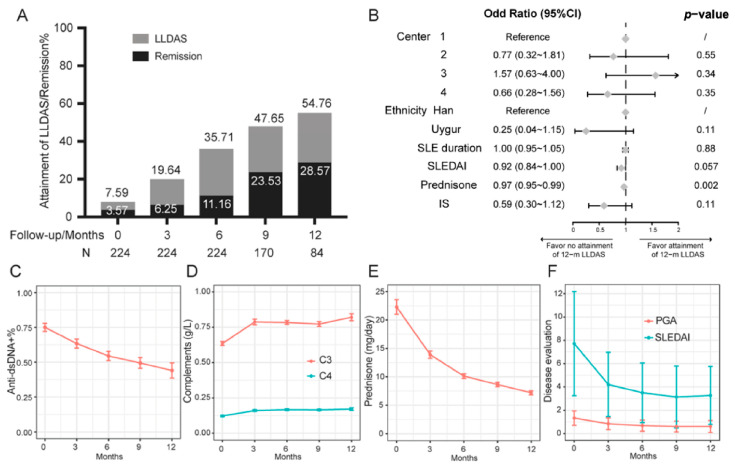
Attainment of LLDAS and remission (**A**). Forrest map (**B**). Changes of individual parameters during follow-up (**C**–**F**).

LLDAS and remission attainment was analyzed as the primary endpoint for all the patients. At baseline, only 7.59% and 3.57% of the patients fulfilled the criteria of LLDAS and remission, respectively. More patients attained this endpoint over time; by 12 months (*n* = 80), 54.76% and 28.57% of patients achieved LLDAS and remission, respectively (Figure 2A). Subgroup analysis according to binary logistic regression indicated that lower prednisone dose at baseline were associated with 12-month LLDAS after adjusting for study center, ethnicity, SLE duration, SLEDAI, and IS (Figure 2B). For separated parameters, SLEDAI score and PGA decreased over time (SLEDAI from 7.72 ± 4.46 to 3.31 ± 2.48; PGA from 1.34 ± 0.62 to 0.61 ± 0.51); the positivity of anti-ds-DNA antibody was reduced from 75.4% to 43.5% as complement levels increased. Daily prednisone was reduced significantly from 23.65 ± 19.70 mg at baseline to 7.32 ± 3.29 mg at 12 months (Figure 2C–F). Of note, only 3 patients discontinued prednisone till the last visit.

**Figure 3:**
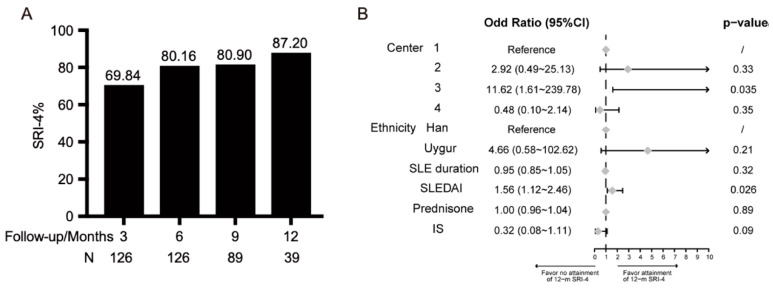
SRI-4 response of patients with moderate/severe disease during follow-up (**A**) and forrest map for sub-analyses (**B**).

For the two subgroups divided by SLEDAI score (SLEDAI score > 6 versus SLEDAI score ≤ 6 (Table 1), 126 patients were in the moderate/severe subgroup with a mean SLEDAI score of 10.68 ± 3.63. Their disease duration was 6.61 ± 5.41 years. They were taking a daily prednisone dose of 31.12 ± 21.61 mg and 65.9% of patients received IS. The 3-month SRI-4 was 69.84% (*n* = 126), and the rate reached over 80% from 6 months to 12 months (Figure 3A). In order to identify predictors of 12-month SRI-4 attainment, forest map (Figure 3B) according to the binary logistic regression indicated that a high baseline SLEDAI was an independent predictor (HR 1.56, 95% CI 1.12~2.46) adjusting study center, ethnicity, SLE duration, SLEDAI, daily prednisone, and IS. In addition, Center 3 had the highest rate of SRI-4 response.

**Figure 4:**
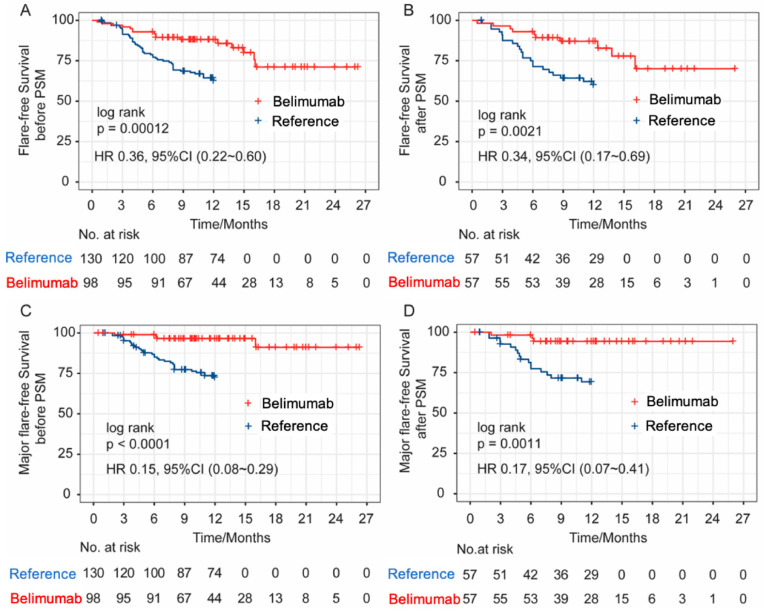
Flare-free (**A**,**B**) and major flare-free (**C**,**D**) survival curves before and after PSM.

For the mild disease subgroup, 98 patients with SLEDAI score of 3.91 ± 1.64 and PGA of 0.96 ± 0.51 were included. Their disease duration was longer than that of the moderate/severe disease subgroup (8.98 ± 7.06 years), and they were taking less daily prednisone (14.77 ± 11.70 mg). The ethnicity distribution was similar between the two subgroups. SFI was evaluated and compared with previous randomized trial control arm as reference (Table S2). Patients taking belimumab were younger (32.63 ± 12.41 vs. 34.23 ± 9.8, *p* = 0.047), had a longer disease duration (8.98 ± 7.06 vs. 4.64 ± 4.77, *p* < 0.001), and had a higher SLEDAI (3.91 ± 1.64 vs. 3.01 ± 2.63, *p* < 0.001). Baseline treatment including prednisone, HCQ, and IS exposure were similar in the two groups. Flare rates were significantly higher in the reference group (16.3% vs. 36.2%, *p* = 0.001). After propensity score matching (PSM) adjusted for age, disease duration, baseline SLEDAI, and prednisone, 57 matched patients from each group were identified. The flare rates were still significantly lower in the belimumab group than those in the reference group after PSM (17.5%, vs. 38.6%, *p* = 0.021). Flare-free survival curves before and after PSM also demonstrated that patients receiving belimumab add on had a significantly lower risk of disease flare and major flare than patients receiving SoC alone (Figure 4) (flare: before PSM (A), HR 0.368, 95% CI (0.225 to 0.603); after PSM (B), HR 0.348, 95% CI (0.173 to 0.700); major flare: before PSM (C), HR 0.15, 95% CI (0.08 to 0.29); after PSM, (D), HR 0.17, 95% CI (0.07 to 0.41)).

## 8. Adverse Events and Treatment Discontinuation

Forty (17.9%) patients had adverse events (AEs) reported (Table 2). Two patients experienced mild allergic reactions and another two patients experienced leukopenia after belimumab infusion. One patient complicated with plane warts and 3 patients with pregnancy during follow-up discontinued the treatment. All the other AEs were infections, most of which were non-invasive, including 12 upper respiratory infections, 5 lower urinary tract infections, 1 herpes simplex virus infections, and 6 herpes zoster reactivations. The remaining 8 patients had invasive infections, of which 2 had gastroenteritis and 6 had pneumonia. In particular, one pneumonia patient was due to invasive fungal infection. Another patient had pulmonary tuberculosis infection after 6 infusions of belimumab, despite of negative baseline screening tests of T-SPOT TB and chest CT scan. All patients recovered after anti-microbial therapy. No HBV reactivation was observed.

Six patients discontinued the treatment with belimumab due to adverse events. Other reasons for discontinuation included lack of effectiveness (*n* = 21), financial/insurance reasons (*n* = 8), COVID-19 endemic situation (*n* = 5, none of them actually had COVID-19 infection), conception plan (*n* = 2), lost to follow-up (*n* = 7), and patients’ will (*n* = 36).

## 9. Discussion

In this multicenter observational study, we demonstrated the effectiveness of belimumab in SLE patients in clinical practice. To the best of our knowledge, this is the first real-world study of belimumab in SLE patients in China. It was confirmed that belimumab add-on to SoC in a mean follow-up of 1 year was effective in the Chinese population in terms of reducing disease activity (SLEDAI and PGA), improving serologic markers, and facilitating glucocorticoid tapering, which was in line with real-life observational studies in other populations [7,10]. Furthermore, belimumab helped lupus patients in achieve LLDAS/remission (54.76% in LLDAS and 28.57% in remission at 12 months), which were key outcome measurements for the treat-to-target strategy in SLE [15,20]. Baseline lower levels of SLEDAI and prednisone were significantly associated with 12-month LLDAS attainment, which was compatible with the results from the SLICC cohort [21].

As an FDA-approved drug for lupus patients with active disease activity (SLEDAI > 6), the efficacy of belimumab has been extensively studied in these patients, nevertheless, it is a legitimate question to ask if belimumab is helpful as a maintenance therapy in patients with low-grade disease activity or in remission. They still have a high risk of disease flares [12], which will lead to further or new organ damage, therefore, an unmet need exists in these patients in clinical practice. In real life, it was a shared decision-making process between investigators and patients by using belimumab among such conditions, with the rationale to reduce lupus flare and spare glucocorticoids. In regard to endpoints, reducing disease activity is a therapeutic goal for active patients. For an example, SRI-4 was a frequently-used endpoint measurement designed to address the efficacy of belimumab in patients with active lupus. However, SRI-4 is not suitable for patients with lower disease activities. Therefore, we subdivided our patients into two groups (SLEDAI score > 6 vs. ≤6). The SRI-4 was used to gauge its effectiveness in patients with moderate/severe disease, whereas the flare index (SFI) was applied as a surrogate outcome measure for those with mild disease. It is not surprising that a high SRI-4 response rate was observed among the moderate/severe disease subgroup. Furthermore, to identify independent factors related with achieving this treatment response, we adjusted possible confounders including study centers, ethnicity, SLE duration, disease activity (SLEDAI), and baseline treatments (daily prednisone dosage and concomitant immunosuppressive therapy). It was identified that a higher SLEDAI score at baseline was an independent predictor of achieving SRI-4, which was replicable in a previous pooled analysis of BLISS trials and other real-life studies [7,22,23]. Of note, SRI-4 response was the highest in Center 3, which was likely explained by the higher SLEDAI at baseline of the enrolled population in this center. However, we failed to replicate other possible predictors, such as shorter disease duration or lower baseline damage index, probably due to limited sample size and shorter follow-up time. Talking about the efficacy of belimumab for lupus patients with different organ involvements, lupus nephritis is an approved indication of belimumab. In this real-world study, 33.5% of patients had active lupus nephritis at baseline, we are enrolling more patients with a longer follow-up (ideally ~2 years) in order to come up with more convincing evidence in this population. Other outcomes related to organs, such as CLASI for skin and DAS28 for joints, were not available in this study.

For the mild subgroup, controls in parallel are lacking in the real-world setting. In order to evaluate the efficacy of belimumab in these patients with the endpoint of reducing disease flares according to SFI, we introduced subjects with low grade disease activity (SLEDAI ≤ 6) in the control arms treated with SoC from our previous metformin randomized trials as reference group [12,19] and the signal of flare risk reduction was detected. After PSM with relevant confounders including age, disease duration and SLEDAI score, there was a 2-fold decrease of flare events among mild patients treated with belimumab. Nevertheless, the result of a randomized placebo-controlled trial is pending [24], to provide a real answer to this question.

Belimumab was overall well tolerated in Chinese lupus patients. Allergic reactions were only observed in two patients. Most adverse events were mild and tolerable, and only 6 patients discontinued the treatment due to adverse events. However, infection events, particularly viral infections and pneumonia were recorded in 7 and 6 patients, respectively. No HBV reactivation was reported in the context of a high prevalence of HBV in the Chinese general population. Similarly, only one TB infection was recorded. Nevertheless, discontinuation was common in our study for various reasons including lack of effectiveness, financial/insurance reasons, COVID-19 endemic situation, conception plan, lost to follow-up, and patients’ will, which is in concordance with some other real-world studies [7,8]. Therefore, overinterpretation should be avoided due to the attrition issue of our observational study design.

The study has several limitations. First, the mean follow-up since the initiation of belimumab was only 1 year; longer exposure with other outcomes evaluation the efficacy to organ involvements and damage accrual endpoint (SLICC/ACR damage index, SDI) [25] is warranted. Second, data attrition and the use of imputation may introduce some bias that needs careful interpretation. Third, randomized, placebo-controlled trials are still needed to address specific questions, such as the flare-prevention issue for patients with mild disease.

## Figures and Tables

**Table 1:** Baseline characteristics of patients in this real-world study.

	Items	All (*n* = 224)	Moderate/Severe (*n* = 126)	Mild (*n* = 98)	*p*	Missing %
**Demographic features**	**Gender (%)**	213 (95.1)	123 (97.6)	90 (91.8)	0.062	0
	**Age (y)**	34.09 (10.91)	33.98 (11.74)	34.23 (9.80)	0.422	0
	**Han**	211 (94.20)	117 (92.86)	94 (95.92)	0.398	0
	**Uygur**	13 (5.80)	9 (7.14)	4 (4.08)	0.398	0
	**SLE duration (y)**	7.65 (6.29)	6.61 (5.41)	8.98 (7.06)	0.014	0
	**SLE duration ≤ 2 y (%)**	65 (29.0)	43 (34.1)	22 (22.4)	0.075	0
	**Follow up (m)**	11.72 (5.11)	10.94 (4.69)	12.73 (5.46)	0.011	0
**Laboratory results**	**Anti-dsDNA+ (%)**	169 (75.4)	105 (83.3)	64 (65.3)	0.003	11.6
	**Complement 3 (g/L)**	0.63 (0.21)	0.58 (0.18)	0.70 (0.22)	<0.001	4.9
	Low C3 (%) *	148 (66.1)	95 (75.4)	53 (54.1)	0.001	4.9
	**Complement 4 (g/L)**	0.12 (0.09)	0.11 (0.09)	0.13 (0.08)	0.036	6.3
	Low C4 (%) *	101 (45.1)	63 (50.0)	38 (38.8)	0.124	6.3
	**IgG (g/L)**	13.36 (5.24)	13.38 (5.58)	13.33 (4.81)	0.984	17.9
**Organ involvement**	**Mucocutaneous**	60 (26.8)	43 (34.1)	17 (17.3)	0.006	0
	**Musculoskeletal**	47 (21.0)	30 (23.8)	17 (17.3)	0.252	0
	**Renal**	75 (33.5)	61 (48.4)	14 (14.3)	<0.001	0
	**Hematologic**	38 (17.0)	27 (21.4)	11 (11.2)	0.049	0
	**Neurological**	5 (2.2)	4 (3.2)	1 (1.0)	0.389	0
**Disease evaluation**	**SLEDAI**	7.72 (4.46)	10.68 (3.63)	3.91 (1.64)	<0.001	0
	**SDI**	0.49 (0.72)	0.48 (0.70)	0.50 (0.75)	0.858	0.9
	**PGA**	1.34 (0.62)	1.62 (0.55)	0.96 (0.51)	<0.001	0.4
**Baseline Therapy**	**Prednisone (mg/d)**	23.97 (19.68)	31.12 (21.61)	14.77 (11.70)	<0.001	0
	**HCQ (%)**	198 (88.4)	113 (89.7)	85 (86.7)	0.533	0
	**IS (%)**	134 (59.8)	83 (65.9)	51 (52.0)	0.040	0
	**MMF (%)**	69 (30.8)	41 (32.5)	28 (28.6)	/	0
	**CTX (%)**	13 (5.8)	12 (9.5)	1 (1.0)	/	0
	**CNI (%)**	43 (19.2)	26 (20.6)	17 (17.4)	/	0
	**Others (%)**	9 (4.02)	4 (3.1)	5 (5.1)	/	0

* C3/C4: complement 3/4.

**Table 2:** Adverse events.

Adverse Events	n (%)
Allergic reaction	2 (0.9)
Leukopenia	2 (0.9)
Plane warts	1 (0.4)
Upper respiratory infection	12 (5.4)
Herpes zoster infections	6 (2.7)
Urinary tract infection	5 (2.2)
HSV infections	1 (0.4)
Pneumonia *	6 (2.7)
Gastroenteritis	2 (0.9)
Pregnancy	3 (1.3)

* Pneumonia: one had invasive fungal infection, one had pulmonary tuberculosis infection and the remaining 4 had bacterial pneumonia.

## Data Availability

Deidentified participants’ data will be available upon reasonable request to the corresponding author.

## Supplementary Materials

The following supporting information can be downloaded at: https://www.mdpi.com/article/10.3390/biomedicines11030962/s1, Table S1: Baseline characteristics of patients in this real-world study from four centers; Table S2. Baseline characteristics of mild disease subgroup and “reference group” from previous randomized trials before and after PSM.
