# Supplementary material for: Effectiveness and Safety of Belimumab in Chinese Lupus Patients: A Multicenter, Real-World Observational Study
Source: Biomedicines. 2023 Mar 21;11(3):962. doi: 10.3390/biomedicines11030962 (PMC10046515; doi:10.3390/biomedicines11030962)
Supplement: Supplementary file 1 [file biomedicines-11-00962-s001.zip › biomedicines-2169318-supplementary.pdf]

Supplementary Materials

Effectiveness and safety of belimumab in Chinese lupus patients: a multicenter, real-world observational study

Fangfang Sun[1], Zitao Wang[2], Tong Wu[3], Xue Wu[4], Jie Chen[1], Danting Zhang[1], Chunde Bao[1], Nan Shen[1], Huaxiang Wu[2], Jing Zhu[3], Lijun Wu[4,5], Shuang Ye[1]

1. Department of Rheumatology, Renji Hospital, School of Medicine, Shanghai Jiaotong University, Shanghai, China.
2. Department of Rheumatology, The Second Affiliated Hospital Zhejiang University School of Medicine, Zhejiang, China.
3. Department of Rheumatology, Sichuan Provincial People's Hospital, University of Electronic Science and Technology of China, Chengdu, China.
4. Department of Rheumatology, People's Hospital of Xinjiang Uygur Autonomous Region, Xinjiang, China
5. Xinjiang Clinical Research Center for Rheumatoid arthritis, Xinjiang, China

Fangfang Sun and Huaxiang Wu contributed equally to this work.

Corresponding authors: Jing Zhu, Lijun Wu.

Table S1. Baseline characteristics of patients in this real-world study from four centers.

	Items	Center 1 (n=52)	Center 2 (n=48)	Center 3 (n=52)	Center 4 (n=72)
Demographic features	Gender (%)	50 (96.2)	44 (91.7)	50 (96.2)	69 (95.8)
	Age (y)	35.54 (11.42)	31.33 (9.44)	33.90 (10.00)	35.03 (11.92)
	Han	52 (100)	48 (100)	52 (100)	59 (81.9)
	Uygur	0 (0)	0 (0)	0 (0)	13 (18.1)
	SLE duration (y)	10.71 (6.34)	8.84 (5.97)	5.71 (4.67)	6.03 (6.54)
	SLE duration≤2 (%)	7 (13.5)	6 (12.5)	15 (28.8)	37 (51.4)
	Follow up (m)	13.29 (5.87)	12.27 (5.33)	10.35 (4.53)	11.22 (4.48)
Laboratory results	Anti-dsDNA+%	35 (67.3)	34 (70.8)	42 (80.8)	58 (80.6)
	Complement 3 (g/L)	0.66 (0.20)	0.67 (0.25)	0.61 (0.19)	0.59 (0.19)
	Complement 4 (g/L)	0.12 (0.08)	0.10 (0.05)	0.12 (0.10)	0.13 (0.09)
	IgG (g/L)	12.63 (4.35)	15.03 (4.71)	12.81 (4.66)	13.18 (6.32)
Organ involvement	Mucocutaneous	8 (15.4)	18 (37.5)	21 (40.4)	13 (18.1)
	Musculoskeletal	4 (7.7)	16 (33.3)	12 (23.1)	15 (20.8)
	Renal	17 (32.7)	9 (18.8)	27 (51.9)	22 (30.6)
	Hematologic	6 (11.5)	5 (10.4)	18 (34.6)	9 (12.5)
	Neurological	2 (3.8)	1 (2.1)	0 (0)	2 (2.8)
Disease evaluation	SLEDAI	6.75 (3.96)	6.44 (4.70)	10.69 (4.54)	7.12 (3.63)
	SDI	0.63 (0.89)	0.31 (0.47)	0.27 (0.53)	0.65 (0.79)
	PGA	1.12 (0.66)	1.36 (0.67)	1.63 (0.63)	1.26 (0.46)
Baseline Therapy	Prednisone (mg/d)	17.46 (11.34)	22.08 (24.61)	30.50 (22.08)	25.21 (17.50)
	HCQ (%)	50 (96.2)	38 (79.2)	45 (86.5)	65 (90.3)

	**IS (%)**	29 (55.8)	32 (66.7)	46 (88.5)	27 (37.5)
	**MMF (%)**	21 (40.4)	14 (29.2)	19 (36.5)	15 (20.8)
	**CTX (%)**	0 (0.0)	0 (0.0)	6 (11.5)	7 (9.7)
	**CNI (%)**	5 (9.6)	17 (35.4)	16 (30.8)	5 (6.9)
	**Others (%)**	3 (5.8)	1 (2.1)	5 (9.6)	0 (0)

Table S2. Baseline characteristics of mild disease subgroup and "reference group" from previous randomized trials before and after PSM.

	**Before PSM**			**After PSM**		
**All patients**	**Belimumab (n=98)**	**Reference (n=130)**	**p**	**Belimumab (n=57)**	**Reference (n=57)**	**p**
**Gender (%)**	90 (91.8)	122 (93.8)	0.607	51 (89.5)	54 (94.7)	0.490
**Age (y)**	34.23 (9.80)	32.63 (12.41)	0.047	31.81 (9.66)	31.75 (10.45)	0.792
**SLE duration (y)**	8.98 (7.06)	4.64 (4.77)	<0.001	6.07 (4.74)	5.47 (4.87)	0.431
**Anti-dsDNA+%**	64 (65.3)	71 (59.2)	0.274	36 (63.2)	36 (63.2)	1.000
**Complement 3 (g/L)**	0.70 (0.22)	0.79 (0.23)	0.001	0.72 (0.23)	0.75 (0.20)	0.300
**Complement 4 (g/L)**	0.13 (0.08)	0.14 (0.08)	0.309	0.13 (0.09)	0.13 (0.07)	0.556
**SLEDAI**	3.91 (1.64)	3.01 (2.63)	<0.001	3.54 (1.69)	3.96 (2.83)	0.971
**Prednisone (mg/d)**	14.77 (11.70)	12.12 (7.60)	0.412	12.89 (10.26)	13.43 (6.28)	0.125
**HCQ (%)**	85 (86.7)	114 (88.4)	0.690	48 (84.2)	48 (84.2)	1.000
**IS (%)**	51 (52.0)	80 (61.5)	0.177	30 (52.6)	36 (63.2)	0.343
**Flare**	16 (16.3)	47 (36.2)	0.001	10 (17.5)	22 (38.6)	0.021
**Major Flare**	4 (4.1)	32 (24.6)	<0.001	3 (5.3)	16 (28.1)	0.0026
